# LINC00963 facilitates acute myeloid leukemia development by modulating miR-608/MMP-15

**DOI:** 10.18632/aging.103252

**Published:** 2020-10-04

**Authors:** Wenli Zuo, Keshu Zhou, Mei Deng, Quande Lin, Qingsong Yin, Chunlei Zhang, Jian Zhou, Yongping Song

**Affiliations:** 1 Department of Hematology, Affiliated Cancer Hospital of Zhengzhou University, Zhengzhou 450008, Henan, China

**Keywords:** acute myeloid leukemia, LINC00963, MMP-15, miR-608

## Abstract

Despite continuous improvements of AML therapy, the prognosis of AML patients remains unsatisfactory. Recently, lncRNAs have been reported to participate in the development of AML. Our data demonstrated that MMP15 and LINC00963 were upregulated and miR-608 was decreased in AML cells (THP-1, HL-60, HEL and MOLM-13) compared to HS-5 cells. RT-qPCR results showed that LINC00963 levels were higher in the serum and bone marrow of AML cases than in controls. Moreover, overexpression of LINC00963 promoted AML cell growth and EMT progression in both THP-1 and HL-60 cells. Furthermore, miR-608 levels were downregulated in the serum and bone marrow of AML cases compared with controls, and Pearson’s correlation analysis indicated that LINC00963 was negatively correlated with miR-608 in the serum and bone marrow of AML samples. In addition, we demonstrated that LINC00963 sponged miR-608 expression and that MMP-15 was a target of miR-608 in AML cells. Finally, rescue experiments indicated that ectopic expression of LINC00963 accelerated cell growth and EMT development by modulating MMP-15. These data demonstrated that LINC00963 acted as an oncogene and may be a potential target for AML treatment.

## INTRODUCTION

Acute myeloid leukemia (AML) remains the most prevalent hematological tumor [1–3]. AML is characterized by impaired interference and apoptosis, uncontrolled proliferation and differentiation in leukemic cells [4–6]. Several risk factors, including age, white blood cell count, cytogenetic risk and gene mutations, have been reported to be related to AML development [5, 7–9]. Despite progress in the treatment of AML, the overall survival rate is still unsatisfactory [10–12]. Thus, there is a need to identify novel molecular mechanisms and treatment targets for AML.

LncRNAs are widely found in mammals and are a group of noncoding RNA transcripts longer than 200 nt with little or no protein-coding ability [13–18]. Growing studies have illustrated that lncRNAs can act as oncogenes or cancer suppressor genes in most tumors, including osteosarcoma, glioblastoma, gastric tumor, gallbladder carcinoma and AML [19–24]. Studies have suggested that lncRNAs play vital roles in a variety of cell activities, such as cell metabolism, invasion, differentiation and migration [25–30]. Recently, LINC00963, a newly discovered lncRNA, has been reported to be involved in the development of several tumors, such as breast cancer, ovarian tumors, esophageal tumors, cutaneous carcinoma, melanoma, hepatocellular carcinoma and prostate cancer [31–37]. However, the clinical relevance and cell function of LINC00963 in AML are still poorly characterized.

We first measured LINC00963 in AML cells, and RT-qPCR analysis demonstrated that LINC00963 was upregulated in AML cells (THP-1, HL-60, HEL and MOLM-13) compared to HS-5 cells. RT-qPCR analysis showed that LINC00963 levels were higher in the serum and bone marrow of AML cases than in controls. Furthermore, overexpression of LINC00963 promoted AML cell growth and EMT progression in both THP-1 and HL-60 cells.

## RESULTS

### MMP15, LINC00963 and miR-608 levels in AML cells

**Figure 1:**
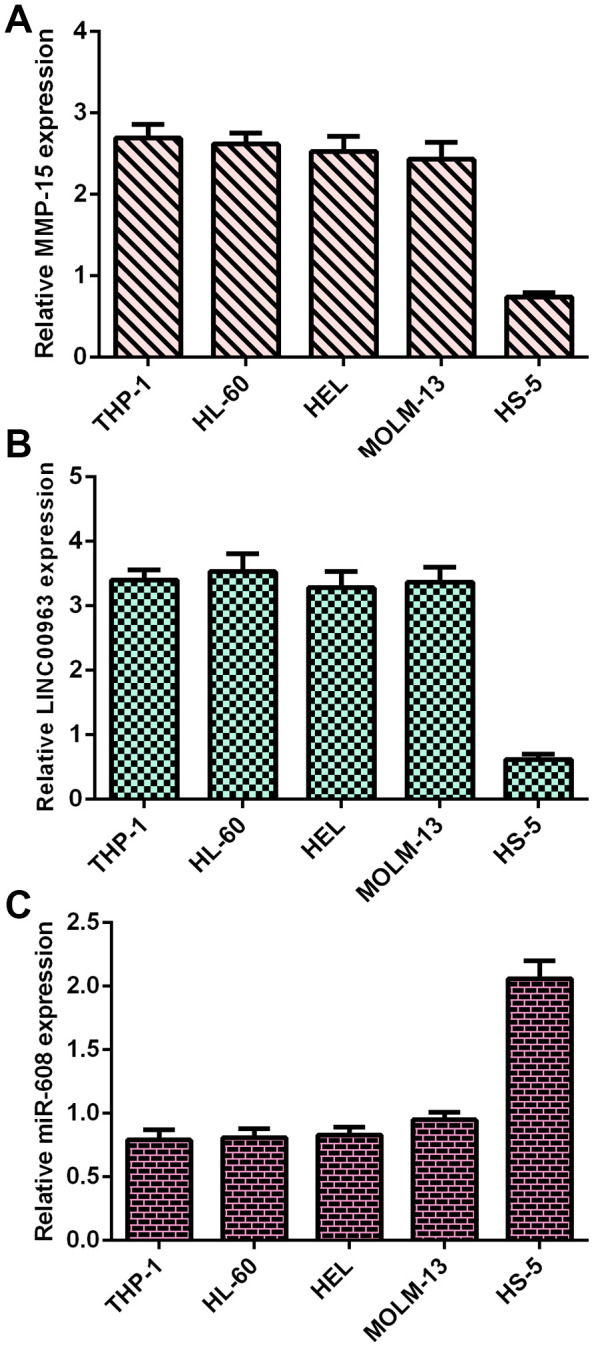
**MMP15, LINC00963 and miR-608 levels in AML cells.** (**A**) MMP15 was overexpressed in AML cells (THP-1, HL-60, HEL and MOLM-13) compared with HS-5 cells. GAPDH was used as the internal control. (**B**) The expression of LINC00963 was determined by RT-qPCR analysis. GAPDH was used as the internal control. (**C**) miR-608 was decreased in AML cells (THP-1, HL-60, HEL and MOLM-13) compared to HS-5 cells. U6 was used as the internal control for miR-608.

Frist, RT-qPCR analysis was utilized to determine MMP15, LINC00963 and miR-608 levels in AML cells. MMP15 was overexpressed in AML cells (THP-1, HL-60, HEL and MOLM-13) compared with HS-5 cells (Figure 1A). LINC00963 was upregulated in AML cells (THP-1, HL-60, HEL and MOLM-13) compared to HS-5 cells (Figure 1B). Moreover, miR-608 was decreased in AML cells (THP-1, HL-60, HEL and MOLM-13) compared to HS-5 cells (Figure 1C).

### LINC00963 level in AML specimens

**Figure 2:**
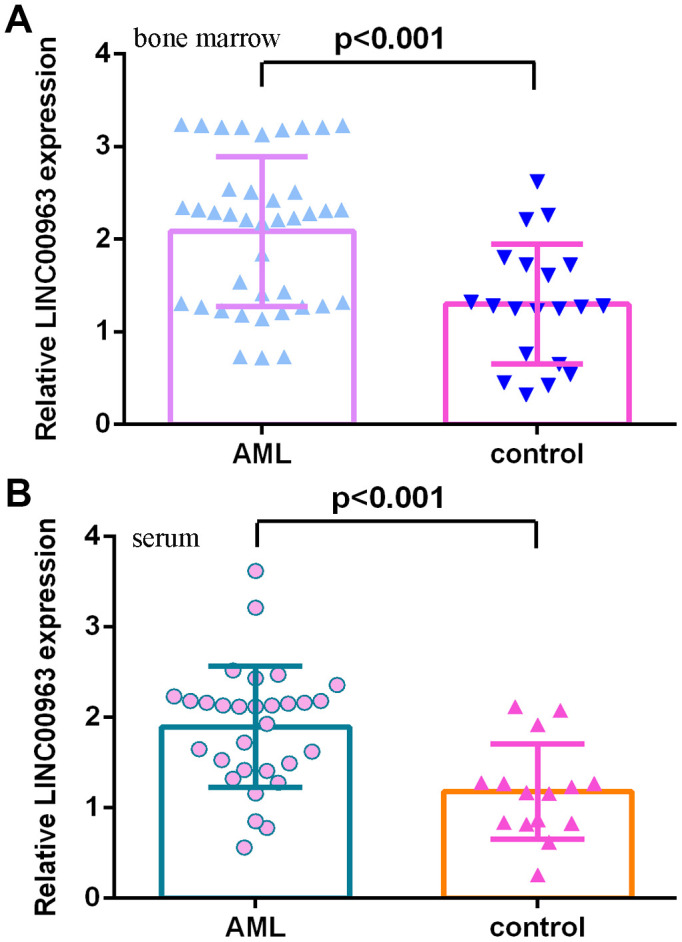
**LINC00963 level in AML specimens.** (**A**) LINC00963 levels were higher in the bone marrow of AML cases than in controls. (**B**) The expression of LINC00963 in the serum of AML cases and controls was measured by RT-qPCR. GAPDH was used as the internal control.

Then, we measured LINC00963 in the serum and bone marrow of AML cases and controls. The data illustrated that LINC00963 levels were higher in the bone marrow of AML cases than in controls (Figure 2A). Moreover, LINC00963 levels were upregulated in the serum of AML cases compared with controls (Figure 2B).

### miR-608 level in AML specimens

**Figure 3:**
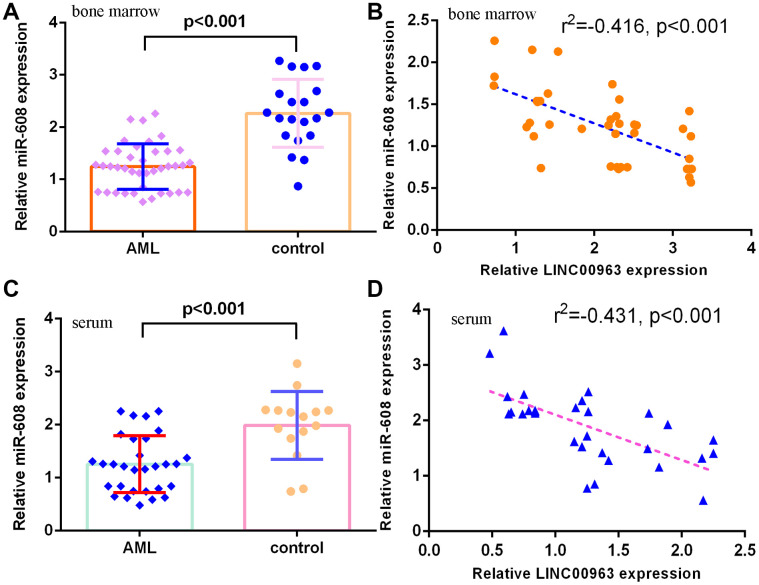
**miR-608 levels in AML specimens.** (**A**) The expression of miR-608 in the bone marrow of AML cases and controls was measured by RT-qPCR. (**B**) Pearson’s correlation analysis indicated that LINC00963 was negatively correlated with miR-608 in the bone marrow of AML samples. (**C**) miR-608 levels were downregulated in the serum of AML cases compared with controls. (**D**) Pearson’s correlation analysis sowed that LINC00963 was negatively correlated with miR-608 in the serum of AML samples. U6 was used as the internal control.

Furthermore, we measured miR-608 in the serum and bone marrow of AML cases and controls. The results demonstrated that miR-608 levels were lower in the bone marrow of AML cases than in controls (Figure 3A). Pearson’s correlation analysis indicated that LINC00963 was negatively correlated with miR-608 in the bone marrow of AML samples (Figure 3B). Moreover, miR-608 levels were downregulated in the serum of AML cases compared with controls (Figure 3C). Pearson’s correlation analysis showed that LINC00963 was negatively correlated with miR-608 in the serum of AML samples (Figure 3D).

### Overexpression of LINC00963 promoted AML cell growth and EMT progression

**Figure 4:**
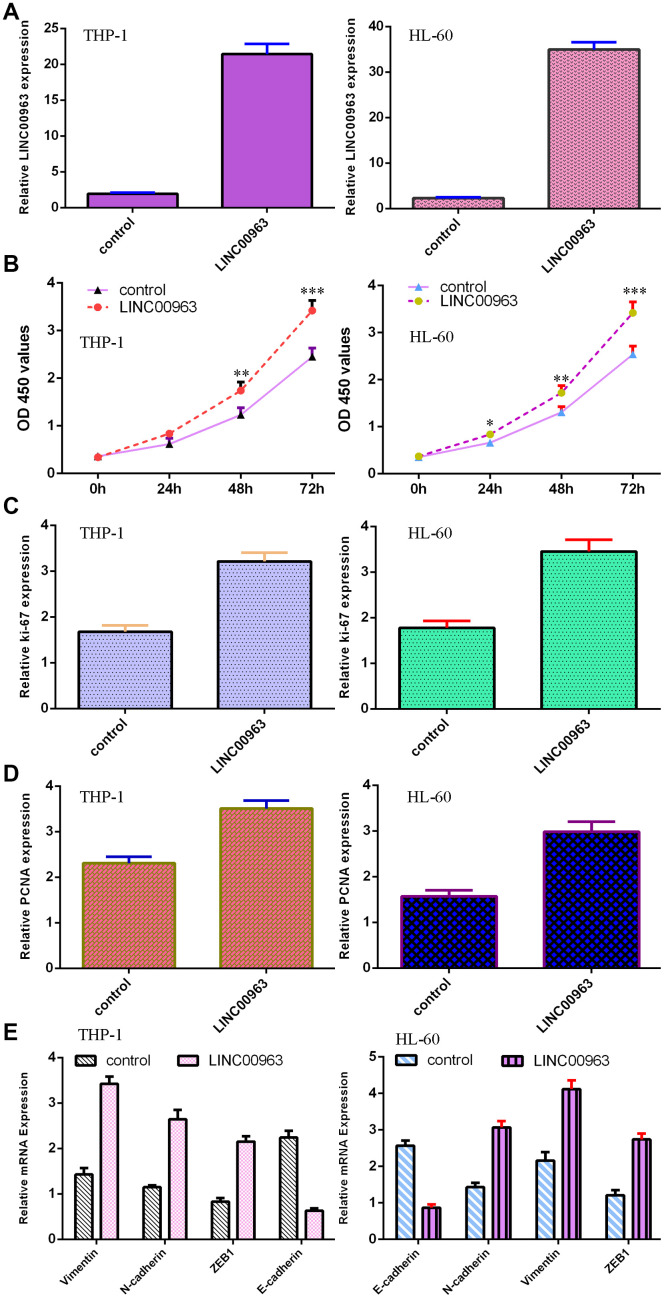
**Overexpression of LINC00963 promoted AML cell growth and EMT progression.** (**A**) The expression of LINC00963 was measured by RT-qPCR in both THP-1 and HL-60 AML cells. (**B**) CCK-8 assay showed that overexpression of LINC00963 accelerated cell growth in both THP-1 and HL-60 cells. (**C**) Elevated expression of LINC00963 facilitated ki-67 expression in both THP-1 and HL-60 cells. (**D**) RT-qPCR assay indicated that elevated expression of LINC00963 accelerated PCNA levels in both THP-1 and HL-60 cells. (**E**) Ectopic expression of LINC00963 enhanced Vimentin, N-cadherin and ZEB1 expression and decreased E-cadherin expression in both THP-1 and HL-60 cells. *p<0.05, **p<0.01 and ***p<0.001. GAPDH was used as the internal control.

The level of LINC00963 was significantly upregulated in both AML cell lines THP-1 and HL-60 after treatment with pcDNA-LINC00963 (Figure 4A). The CCK-8 assay indicated that overexpression of LINC00963 accelerated cell growth in both THP-1 and HL-60 cells (Figure 4B). Elevated expression of LINC00963 upregulated ki-67 expression in both THP-1 and HL-60 cells (Figure 4C). RT-qPCR assays illustrated that elevated expression of LINC00963 increased PCNA levels in both THP-1 and HL-60 cells (Figure 4D). Moreover, ectopic expression of LINC00963 enhanced Vimentin, N-cadherin and ZEB1 expression and decreased E-cadherin expression in both THP-1 and HL-60 cells (Figure 4E).

### LINC00963 inhibited miR-608 expression in AML cells

**Figure 5:**
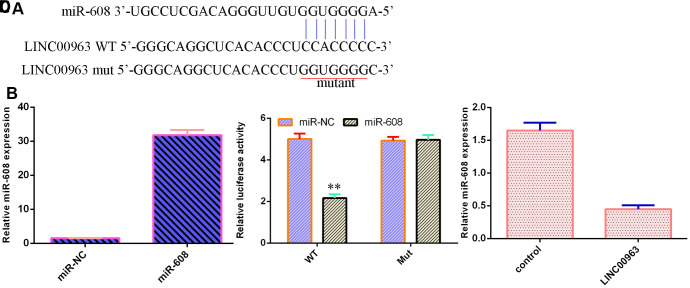
**LINC00963 inhibited miR-608 expression in AML cells.** (**A**) An online database (starBase) predicted that miR-608 was a potential target of LINC00963. (**B**) The level of miR-608 was determined by RT-qPCR. (**C**) Luciferase reporter assays verified that elevated expression of miR-608 decreased the luciferase value in the LINC00963-wt group but did not change the luciferase value in the LINC00963-mut group. (**D**) Overexpression of LINC00963 suppressed miR-608 levels in THP-1 cells. **p<0.01. U6 was used as the internal control.

An online database (starBase) predicted that miR-608 was a potential target of LINC00963 (Figure 5A). The level of miR-608 was significantly upregulated in THP-1 cells after treatment with the miR-608 mimic (Figure 5B). Luciferase reporter assays verified that elevated expression of miR-608 decreased the luciferase value in the LINC00963-wt group but did not change the luciferase value in the LINC00963-mut group (Figure 5C). Overexpression of LINC00963 suppressed miR-608 levels in THP-1 cells (Figure 5D).

### MMP-15 was a target of miR-608

**Figure 6:**
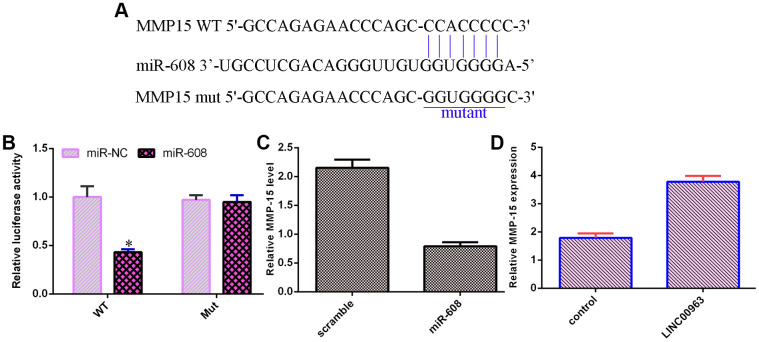
**MMP-15 was a target of miR-608.** (**A**) An online database (TargetScan) predicted that MMP-15 was a potential target of miR-608. (**B**) Luciferase reporter assays verified that elevated expression of miR-608 decreased the luciferase value in the MMP-15-wt group but did not change the luciferase value in the MMP-15-mut group. (**C**) Overexpression of miR-608 suppressed MMP-15 levels in THP-1 cells. (**D**) Ectopic expression of LINC00963 upregulated MMP-15 levels in THP-1 cells. *p<0.05. GAPDH was used as the internal control.

An online database (TargetScan) predicted that MMP-15 was a potential target of miR-608 (Figure 6A). Luciferase reporter analysis verified that elevated expression of miR-608 decreased the luciferase value in the MMP-15-wt group but did not change the luciferase value in the MMP-15-mut group (Figure 6B). Overexpression of miR-608 suppressed MMP-15 levels in THP-1 cells (Figure 6C). Ectopic expression of LINC00963 upregulated MMP-15 levels in THP-1 cells (Figure 6D).

### Ectopic expression of LINC00963 increased cell growth and EMT development by modulating MMP-15

**Figure 7:**
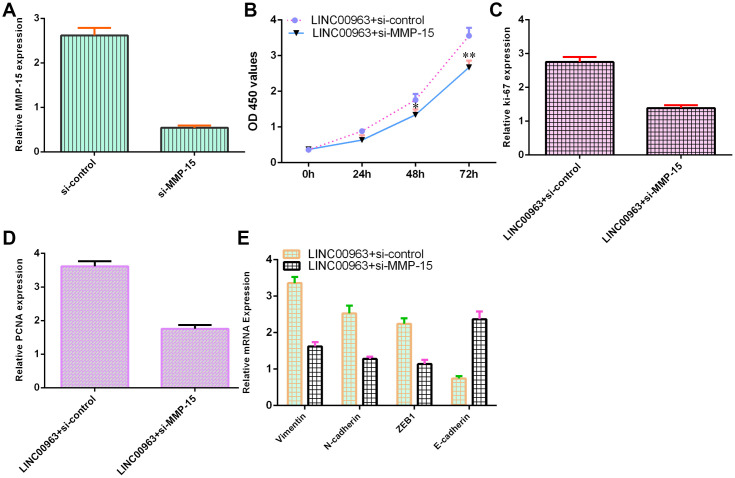
**Ectopic expression of LINC00963 increased cell growth and EMT development by modulating MMP-15.** (**A**) The level of MMP-15 was determined by RT-qPCR. (**B**) CCK-8 assay was performed to detect cell proliferation. (**C**) The level of ki-67 was measured by RT-qPCR. (**D**) The expression of PCNA was measured by RT-qPCR. (**E**) Downregulation of MMP-15 suppressed Vimentin, N-cadherin and ZEB1 levels and upregulated E-cadherin expression in LINC00963-overexpressing THP-1 cells. *p<0.05 and**p<0.01. GAPDH was used as the internal control.

To further study the function of LINC00963 and MMP-15 in AML development, we treated LINC00963-overexpressing THP-1 cells with an siRNA-MMP-15 plasmid. MMP-15 expression was downregulated in THP-1 cells after treatment with the siRNA-MMP-15 plasmid, as demonstrated by RT-qPCR assay (Figure 7A). MMP-15 knockdown inhibited cell growth in LINC00963-overexpressing THP-1 cells (Figure 7B). Knockdown of MMP-15 suppressed ki-67 (Figure 7C) and PCNA levels (Figure 7D) in LINC00963-overexpressing THP-1 cells. Furthermore, downregulation of MMP-15 suppressed Vimentin, N-cadherin and ZEB1 levels and upregulated E-cadherin expression in LINC00963-overexpressing THP-1 cells (Figure 7E).

## DISCUSSION

Despite continuous advances in AML therapy, the prognosis of AML patients remains unsatisfactory [38–40]. Recently, lncRNAs have been reported to participate in the development of AML [5, 19, 41–43]. This research explored the role of LINC00963 in the progression of AML. Our data showed that MMP15 and LINC00963 were upregulated and miR-608 was decreased in AML cells (THP-1, HL-60, HEL and MOLM-13) compared to HS-5 cells. RT-qPCR results demonstrated that LINC00963 levels were higher in the serum and bone marrow of AML cases than in controls. Moreover, overexpression of LINC00963 promoted AML cell growth and EMT progression in both THP-1 and HL-60 cells. Moreover, miR-608 levels were downregulated in the serum and bone marrow of AML cases compared with controls, and Pearson’s correlation analysis indicated that LINC00963 was negatively correlated with miR-608 in the serum and bone marrow of AML samples. In addition, we determined that LINC00963 sponged miR-608 expression and that MMP-15 was a target of miR-608 in AML cells. Finally, rescue experiments indicated that ectopic expression of LINC00963 accelerated cell growth and EMT development by modulating MMP-15. These data illustrated that LINC00963 acted as an oncogene and may be a potential target for AML cases.

Recently, LINC00963, a novel lncRNA, has been reported to be involved in the development of several tumors, such as breast cancer, ovarian tumors, esophageal tumors, cutaneous carcinoma, melanoma, hepatocellular carcinoma and prostate cancer [31–37]. Wu et al [44]. noted that LINC00963 was upregulated in breast tumor specimens, and higher levels of LINC00963 were associated with TNM stage, differentiation grade and lymph node metastasis. Knockdown of LINC00963 suppressed cell invasion and proliferation and accelerated apoptosis, partly regulating miR-625/HMGA1. Liu et al [36]. demonstrated that LINC00963 induced ovarian tumor EMT progression and growth by regulating CHI3L1/miR-378g. Liu et al [35]. noted that LINC00963 enhanced esophageal tumor invasion by modulating RAB14/miR-214-5p. Zhou et al [45]. indicated that LINC00963 accelerated osteosarcoma cell invasion and growth by inhibiting miR-204-3p/FN1. However, the clinical relevance and cell function of LINC00963 in AML are still poorly characterized. We first measured LINC00963 in AML cells, and RT-qPCR analysis illustrated that LINC00963 was upregulated in AML cells (THP-1, HL-60, HEL and MOLM-13) compared to HS-5 cells. Then, we determined LINC00963 levels in the serum and bone marrow of AML cases and controls, and RT-qPCR analysis showed that LINC00963 levels were higher in the serum and bone marrow of AML cases than in controls. Furthermore, overexpression of LINC00963 promoted AML cell growth and EMT progression in both THP-1 and HL-60 cells.

A growing number of lncRNAs have emerged as ceRNAs that protect mRNAs from targeting by miRNAs [46, 47]. A previous study showed that LOC285758 induced AML cell invasion by inhibiting miR-204-5p [48]. Tian et al [5]. noted that lncRNA SBF2-AS1 regulated AML cell growth by regulating miR-188-5p. Gan et al [49]. showed that lncRNA ZFAS1 knockdown decreased AML development by modulating the miR-150/Myb/Sp1 pathway. Dong et al [50]. indicated that HOXA-AS2 knockdown inhibited AML chemoresistance by sponging miR-520c-3p/S100A4. Moreover, Jiao et al [33]. demonstrated that overexpression of LINC00963 facilitated melanoma development by sponging miR-608/NACC1. An online database (starBase) predicted that miR-608 was a potential target of LINC00963. Luciferase reporter assays verified that elevated expression of miR-608 decreased the luciferase value in the LINC00963-wt group but did not change the luciferase value in the LINC00963-mut group. These results indicated that LINC00963 inhibited miR-608 expression in AML cells. Furthermore, we identified MMP-15 as a target gene of miR-608 in AML cells. Wu et al. showed that MMP-15 was overexpressed in AML cells and in peripheral blood and bone marrow of AML. Our study demonstrated that ectopic expression of LINC00963 accelerated cell growth and EMT development by modulating MMP-15.

Our results illustrated that LINC00963 was upregulated in AML cells and that LINC00963 levels were higher in the serum and bone marrow of AML cases than in controls. Ectopic expression of LINC00963 accelerated cell growth and EMT development by modulating the miR-608/MMP-15 axis.

## MATERIALS AND METHODS

### Specimens, cell culture and transfection

Serum and bone marrow samples were obtained from AML patients and control cases at the Affiliated Cancer Hospital of Zhengzhou University. Our research was approved by the Clinical Ethics Committees Board of the Affiliated Cancer Hospital of Zhengzhou University, and written informed consent was obtained from each participant. HS-5 and AML cells (THP-1, HL-60, HEL and MOLM-13) were obtained from ATCC and cultured in RPMI-1640 medium supplemented with antibiotics and FBS. miR-608 mimic, siRNA-MMP-15, pcDNA-LINC00963 and controls were purchased from Genema and transfected using Lipofectamine^TM^2000.

### Cell proliferation assays

Cell growth was determined with a CCK-8 assay (Dojindo, Japan). Cells were cultured in 96-well plates and measured at 0, 1, 2 and 3 days. The cell number was determined from the optical density (OD) at 450 nm.

### RT-qPCR

Total RNA from all specimens and cells was isolated with a TRIzol kit (Ambion, USA). For mRNA, miRNA and lncRNA expression, RT-qPCR was used on an ABI7500 PCR system (Applied Biosystems, USA) using a SYBR kit (TaKaRa, China). The fold change of these genes was determined with the 2^-ΔΔCt^ method. U6 and GAPDH were used as controls. The primer sequences were as follows: LINC00963, 5’-GGTAA ATCGA GGCCC AGAGAT-3’ (F), 5’-ACGTG GATGA CAGCG TGTGA-3’ (R); GAPDH, 5’-CATGA GAAGT ATGAC AACAG CCT-3’ (F), 5’- AGTCC TTCCA CGATAC CAAAGT-3’ (R); miR-608 5’-AGGGG TGGTG TTGGG ACAGC-3’; and U6, 5’-GCGCG TCGTG AAGCG TTC-3’ (F), 5’- GTGCA GGGTC CGAGG -3’ (R).

### Luciferase reporter assay

For the luciferase reporter assay, wild-type LINC00963 and wild-type MMP-15 (LINC00963-wt and MMP-15-wt) or sequences with mutant binding sites (LINC00963-mut and MMP-15-mut) were cloned downstream of the Renilla luciferase gene in the pRLTK vector (Promega, USA). Each vector was cotransfected into cells with miR-608 mimic or control (RiboBio, China). Luciferase analysis was carried out with a dual luciferase detection reagent (Promega, USA), and the relative luciferase value was calculated from Renilla luciferase activity.

### Statistical analysis

The data values are presented as the mean ± SD. Analysis of statistical differences was assessed with Student’s t-test using SPSS 18.0 (Chicago, USA). The significance level was set at P<0.05. Spearman’s correlation analysis was performed to study the correlation.

## References

[1] Foran JM. New prognostic markers in acute myeloid leukemia: perspective from the clinic. Hematology Am Soc Hematol Educ Program. 2010; 2010:47–55. 10.1182/asheducation-2010.1.47 21239770

[2] Fayyad-Kazan H, Bitar N, Najar M, Lewalle P, Fayyad-Kazan M, Badran R, Hamade E, Daher A, Hussein N, ElDirani R, Berri F, Vanhamme L, Burny A, et al. Circulating miR-150 and miR-342 in plasma are novel potential biomarkers for acute myeloid leukemia. J Transl Med. 2013; 11:31. 10.1186/1479-5876-11-31 23391324 PMC3579719

[3] Zhu B, Xi X, Liu Q, Cheng Y, Yang H. MiR-9 functions as a tumor suppressor in acute myeloid leukemia by targeting CX chemokine receptor 4. Am J Transl Res. 2019; 11:3384–97. 31312352 PMC6614627

[4] Peters AH, Schwaller J. Epigenetic mechanisms in acute myeloid leukemia. Prog Drug Res. 2011; 67:197–219. 10.1007/978-3-7643-8989-5_10 21141731

[5] Tian YJ, Wang YH, Xiao AJ, Li PL, Guo J, Wang TJ, Zhao DJ. Long noncoding RNA SBF2-AS1 act as a ceRNA to modulate cell proliferation via binding with miR-188-5p in acute myeloid leukemia. Artif Cells Nanomed Biotechnol. 2019; 47:1730–37. 10.1080/21691401.2019.1608221 31062614

[6] Yi YY, Yi J, Zhu X, Zhang J, Zhou J, Tang X, Lin J, Wang P, Deng ZQ. Circular RNA of vimentin expression as a valuable predictor for acute myeloid leukemia development and prognosis. J Cell Physiol. 2019; 234:3711–19. 10.1002/jcp.27145 30152863

[7] Mayer K, Hahn-Ast C, Schwab K, Schmidt-Wolf IG, Brossart P, Glasmacher A, von Lilienfeld-Toal M. Long-term follow-up of cladribine, high-dose cytarabine, and idarubicin as salvage treatment for relapsed acute myeloid leukemia and literature review. Eur J Haematol. 2020; 104:538–45. 10.1111/ejh.13395 32049382

[8] Chen XX, Lin J, Qian J, Qian W, Yang J, Ma JC, Deng ZQ, Xie D, An C, Tang CY, Qian Z. Dysregulation of miR-124-1 predicts favorable prognosis in acute myeloid leukemia. Clin Biochem. 2014; 47:63–66. 10.1016/j.clinbiochem.2013.09.020 24135052

[9] Vázquez I, Maicas M, Marcotegui N, Conchillo A, Guruceaga E, Roman-Gomez J, Calasanz MJ, Agirre X, Prosper F, Odero MD. Silencing of hsa-miR-124 by EVI1 in cell lines and patients with acute myeloid leukemia. Proc Natl Acad Sci USA. 2010; 107:E167–68. 10.1073/pnas.1011540107 20930122 PMC2973895

[10] Gurnari C, Voso MT, Maciejewski JP, Visconte V. From bench to bedside and beyond: therapeutic scenario in acute myeloid leukemia. Cancers (Basel). 2020; 12:357. 10.3390/cancers12020357 32033196 PMC7072629

[11] Gao XN, Lin J, Li YH, Gao L, Wang XR, Wang W, Kang HY, Yan GT, Wang LL, Yu L. MicroRNA-193a represses C-kit expression and functions as a methylation-silenced tumor suppressor in acute myeloid leukemia. Oncogene. 2011; 30:3416–28. 10.1038/onc.2011.62 21399664

[12] Schwind S, Marcucci G, Maharry K, Radmacher MD, Mrózek K, Holland KB, Margeson D, Becker H, Whitman SP, Wu YZ, Metzeler KH, Powell BL, Kolitz JE, et al. BAALC and ERG expression levels are associated with outcome and distinct gene and microRNA expression profiles in older patients with de novo cytogenetically normal acute myeloid leukemia: a cancer and leukemia group B study. Blood. 2010; 116:5660–69. 10.1182/blood-2010-06-290536 20841507 PMC3031412

[13] Li Z, Li X, Chen X, Li S, Ho IH, Liu X, Chan MT, Wu WK. Emerging roles of long non-coding RNAs in neuropathic pain. Cell Prolif. 2019; 52:e12528. 10.1111/cpr.12528 30362191 PMC6430490

[14] Chen C, Tan H, Bi J, Li L, Rong T, Lin Y, Sun P, Liang J, Jiao Y, Li Z, Sun L, Shen J. LncRNA-SULT1C2A regulates Foxo4 in congenital scoliosis by targeting rno-miR-466c-5p through PI3K-ATK signalling. J Cell Mol Med. 2019; 23:4582–91. 10.1111/jcmm.14355 31044535 PMC6584475

[15] Zou Y, Zhong Y, Wu J, Xiao H, Zhang X, Liao X, Li J, Mao X, Liu Y, Zhang F. Long non-coding PANDAR as a novel biomarker in human cancer: a systematic review. Cell Prolif. 2018; 51:e12422. 10.1111/cpr.12422 29226461 PMC6528858

[16] Zhu S, Fu W, Zhang L, Fu K, Hu J, Jia W, Liu G. LINC00473 antagonizes the tumour suppressor miR-195 to mediate the pathogenesis of Wilms tumour via IKKα. Cell Prolif. 2018; 51:e12416. 10.1111/cpr.12416 29159834 PMC6528909

[17] Zhao J, Zhang C, Gao Z, Wu H, Gu R, Jiang R. Long non-coding RNA ASBEL promotes osteosarcoma cell proliferation, migration, and invasion by regulating microRNA-21. J Cell Biochem. 2018; 119:6461–69. 10.1002/jcb.26671 29323740

[18] Zhang J, Yin M, Peng G, Zhao Y. CRNDE: an important oncogenic long non-coding RNA in human cancers. Cell Prolif. 2018; 51:e12440. 10.1111/cpr.12440 29405523 PMC6528921

[19] Zheng J, Song Y, Li Z, Tang A, Fei Y, He W. The implication of lncRNA expression pattern and potential function of lncRNA RP4-576H24.2 in acute myeloid leukemia. Cancer Med. 2019; 8:7143–60. 10.1002/cam4.2518 31568697 PMC6885877

[20] Sun H, Sun Y, Chen Q, Xu Z. LncRNA KCNQ1OT1 contributes to the progression and chemoresistance in acute myeloid leukemia by modulating Tspan3 through suppressing miR-193a-3p. Life Sci. 2020; 241:117161. 10.1016/j.lfs.2019.117161 31837329

[21] Yang C, Wu K, Wang S, Wei G. Long non-coding RNA XIST promotes osteosarcoma progression by targeting YAP via miR-195-5p. J Cell Biochem. 2018; 119:5646–56. 10.1002/jcb.26743 29384226

[22] Pop S, Enciu AM, Necula LG, Tanase C. Long non-coding RNAs in brain tumours: focus on recent epigenetic findings in glioma. J Cell Mol Med. 2018; 22:4597–610. 10.1111/jcmm.13781 30117678 PMC6156469

[23] Cao C, Xu Y, Du K, Mi C, Yang C, Xiang L, Xie Y, Liu W. LINC01303 functions as a competing endogenous RNA to regulate EZH2 expression by sponging miR-101-3p in gastric cancer. J Cell Mol Med. 2019; 23:7342–48. 10.1111/jcmm.14593 31497936 PMC6815915

[24] Kong L, Wu Q, Zhao L, Ye J, Li N, Yang H. Identification of messenger and long noncoding RNAs associated with gallbladder cancer via gene expression profile analysis. J Cell Biochem. 2019; 120:19377–87. 10.1002/jcb.28953 31498480

[25] Yu X, Zheng H, Tse G, Zhang L, Wu WK. CASC2: an emerging tumour-suppressing long noncoding RNA in human cancers and melanoma. Cell Prolif. 2018; 51:e12506. 10.1111/cpr.12506 30094876 PMC6528875

[26] Xu R, Feng F, Yu X, Liu Z, Lao L. LncRNA SNHG4 promotes tumour growth by sponging miR-224-3p and predicts poor survival and recurrence in human osteosarcoma. Cell Prolif. 2018; 51:e12515. 10.1111/cpr.12515 30152090 PMC6528889

[27] Xiong WC, Han N, Wu N, Zhao KL, Han C, Wang HX, Ping GF, Zheng PF, Feng H, Qin L, He P. Interplay between long noncoding RNA ZEB1-AS1 and miR-101/ZEB1 axis regulates proliferation and migration of colorectal cancer cells. Am J Transl Res. 2018; 10:605–17. 29511455 PMC5835826

[28] She K, Yan H, Huang J, Zhou H, He J. miR-193b availability is antagonized by LncRNA-SNHG7 for FAIM2-induced tumour progression in non-small cell lung cancer. Cell Prolif. 2018; 51:e12406. 10.1111/cpr.12406 29131440 PMC6528931

[29] Pan Y, Wu Y, Hu J, Shan Y, Ma J, Ma H, Qi X, Jia L. Long noncoding RNA HOTAIR promotes renal cell carcinoma Malignancy through alpha-2, 8-sialyltransferase 4 by sponging microRNA-124. Cell Prolif. 2018; 51:e12507. 10.1111/cpr.12507 30105850 PMC6528863

[30] Li Z, Li X, Chen C, Li S, Shen J, Tse G, Chan MT, Wu WK. Long non-coding RNAs in nucleus pulposus cell function and intervertebral disc degeneration. Cell Prolif. 2018; 51:e12483. 10.1111/cpr.12483 30039593 PMC6528936

[31] Wang L, Han S, Jin G, Zhou X, Li M, Ying X, Wang L, Wu H, Zhu Q. Linc00963: a novel, long non-coding RNA involved in the transition of prostate cancer from androgen-dependence to androgen-independence. Int J Oncol. 2014; 44:2041–49. 10.3892/ijo.2014.2363 24691949

[32] Wu JH, Tian XY, An QM, Guan XY, Hao CY. LINC00963 promotes hepatocellular carcinoma progression by activating PI3K/AKT pathway. Eur Rev Med Pharmacol Sci. 2018; 22:1645–52. 10.26355/eurrev_201803_14574 29630107

[33] Jiao H, Jiang S, Wang H, Li Y, Zhang W. Upregulation of LINC00963 facilitates melanoma progression through miR-608/NACC1 pathway and predicts poor prognosis. Biochem Biophys Res Commun. 2018; 504:34–39. 10.1016/j.bbrc.2018.08.115 30180950

[34] Wang J, Li C, Xu L, Yang C, Zhang X. MiR-1193 was sponged by LINC00963 and inhibited cutaneous squamous cell carcinoma progression by targeting SOX4. Pathol Res Pract. 2019; 215:152600. 10.1016/j.prp.2019.152600 31477326

[35] Liu HF, Zhen Q, Fan YK. LINC00963 predicts poor prognosis and promotes esophageal cancer cells invasion via targeting miR-214-5p/RAB14 axis. Eur Rev Med Pharmacol Sci. 2020; 24:164–73. 10.26355/eurrev_202001_19907 31957829

[36] Liu W, Yang YJ, An Q. LINC00963 promotes ovarian cancer proliferation, migration and EMT via the miR-378g / CHI3L1 axis. Cancer Manag Res. 2020; 12:463–73. 10.2147/CMAR.S229083 32021459 PMC6982455

[37] Zhang N, Zeng X, Sun C, Guo H, Wang T, Wei L, Zhang Y, Zhao J, Ma X. LncRNA LINC00963 promotes tumorigenesis and radioresistance in breast cancer by sponging miR-324-3p and inducing ACK1 expression. Mol Ther Nucleic Acids. 2019; 18:871–81. 10.1016/j.omtn.2019.09.033 31751910 PMC6881674

[38] Schwind S, Maharry K, Radmacher MD, Mrózek K, Holland KB, Margeson D, Whitman SP, Hickey C, Becker H, Metzeler KH, Paschka P, Baldus CD, Liu S, et al. Prognostic significance of expression of a single microRNA, miR-181a, in cytogenetically normal acute myeloid leukemia: a cancer and leukemia group B study. J Clin Oncol. 2010; 28:5257–64. 10.1200/JCO.2010.29.2953 21079133 PMC3018359

[39] Langer C, Marcucci G, Holland KB, Radmacher MD, Maharry K, Paschka P, Whitman SP, Mrózek K, Baldus CD, Vij R, Powell BL, Carroll AJ, Kolitz JE, et al. Prognostic importance of MN1 transcript levels, and biologic insights from MN1-associated gene and microRNA expression signatures in cytogenetically normal acute myeloid leukemia: a cancer and leukemia group B study. J Clin Oncol. 2009; 27:3198–204. 10.1200/JCO.2008.20.6110 19451432 PMC2716941

[40] Hackanson B, Bennett KL, Brena RM, Jiang J, Claus R, Chen SS, Blagitko-Dorfs N, Maharry K, Whitman SP, Schmittgen TD, Lübbert M, Marcucci G, Bloomfield CD, Plass C. Epigenetic modification of CCAAT/enhancer binding protein alpha expression in acute myeloid leukemia. Cancer Res. 2008; 68:3142–51. 10.1158/0008-5472.CAN-08-0483 18451139

[41] Chen C, Wang P, Mo W, Zhang Y, Zhou W, Deng T, Zhou M, Chen X, Wang S, Wang C. lncRNA-CCDC26, as a novel biomarker, predicts prognosis in acute myeloid leukemia. Oncol Lett. 2019; 18:2203–11. 10.3892/ol.2019.10591 31452721 PMC6676650

[42] Ke SD, Zhou XF. LncRNA MVIH knockdown inhibits the malignancy progression through downregulating miR-505 mediated HMGB1 and CCNE2 in acute myeloid leukemia. Translational cancer research. 2019; 8:2526 10.21037/tcr.2019.10.12 PMC8799070 35117009

[43] Wang K, Dai J, Liu T, Wang Q, Pang YX. LncRNA ZEB2-AS1 regulates the drug resistance of acute myeloid leukemia via the miR-142-3p/INPP4B axis. Rsc Adv. 2019; 9:39495–504. 10.1039/C9RA07854A PMC9076093 35540690

[44] Wu Z, Wang W, Wang Y, Wang X, Sun S, Yao Y, Zhang Y, Ren Z. Long noncoding RNA LINC00963 promotes breast cancer progression by functioning as a molecular sponge for microRNA-625 and thereby upregulating HMGA1. Cell Cycle. 2020; 5:6100–624. 10.1080/15384101.2020.1728024 32052688 PMC7100992

[45] Zhou Y, Yin L, Li H, Liu LH, Xiao T. The LncRNA LINC00963 facilitates osteosarcoma proliferation and invasion by suppressing miR-204-3p/FN1 axis. Cancer Biol Ther. 2019; 20:1141–48. 10.1080/15384047.2019.1598766 30975024 PMC6605988

[46] Ba Z, Gu L, Hao S, Wang X, Cheng Z, Nie G. Downregulation of lncRNA CASC2 facilitates osteosarcoma growth and invasion through miR-181a. Cell Prolif. 2018; 51:e12409. 10.1111/cpr.12409 29194827 PMC6528952

[47] Tian C, Deng Y, Jin Y, Shi S, Bi H. Long non-coding RNA RNCR3 promotes prostate cancer progression through targeting miR-185-5p. Am J Transl Res. 2018; 10:1562–70. 29887969 PMC5992541

[48] Xue F, Che H. The long non-coding RNA LOC285758 promotes invasion of acute myeloid leukemia cells by down-regulating miR-204-5p. FEBS Open Bio. 2020; 10:734–43. 10.1002/2211-5463.12814 32067385 PMC7193155

[49] Gan S, Ma P, Ma J, Wang W, Han H, Chen L, Li X, Wu F, Sun H. Knockdown of ZFAS1 suppresses the progression of acute myeloid leukemia by regulating microRNA-150/Sp1 and microRNA-150/myb pathways. Eur J Pharmacol. 2019; 844:38–48. 10.1016/j.ejphar.2018.11.036 30502345

[50] Dong X, Fang Z, Yu M, Zhang L, Xiao R, Li X, Pan G, Liu J. Knockdown of long noncoding RNA HOXA-AS2 suppresses chemoresistance of acute myeloid leukemia via the miR-520c-3p/S100A4 axis. Cell Physiol Biochem. 2018; 51:886–96. 10.1159/000495387 30466095

